# Immunoglobulin Free Light Chain Dimers in Human Diseases

**DOI:** 10.1100/tsw.2011.65

**Published:** 2011-03-22

**Authors:** Batia Kaplan, Avi Livneh, Ben-Ami Sela

**Affiliations:** ^1^ Heller Institute of Medical Research, Sheba Medical Center, Tel-Hashomer, Israel; ^2^ Sackler School of Medicine, Tel-Aviv University, Tel-Aviv, Israel; ^3^ Institute of Chemical Pathology, Sheba Medical Center, Tel-Hashomer, Israel

**Keywords:** free light chains, monomers, dimers, Western blotting, amyloidosis, multiple myeloma, multiple sclerosis

## Abstract

Immunoglobulin free light chain (FLC) kappa (κ) and lambda (λ) isotypes exist mainly in monomeric and dimeric forms. Under pathological conditions, the level of FLCs as well as the structure of monomeric and dimeric FLCs and their dimerization properties might be significantly altered. The abnormally high fractions of dimeric FLCs were demonstrated in the serum of patients with multiple myeloma (MM) and primary systemic amyloidosis (AL), as well as in the serum of anephric patients. The presence of tetra- and trimolecular complexes formed due to dimer-dimer and dimer-monomer interactions was detected in the myeloma serum. Analysis of the amyloidogenic light chains demonstrated mutations within the dimer interface, thus raising the possibility that these mutations are responsible for amyloidogenicity. Increased κ monomer and dimer levels, as well as a high κ/λ monomer ratio, were typically found in the cerebrospinal fluid from patients with multiple sclerosis (MS). In many MS cases, the elevation of κ FLCs was accompanied by an abnormally high proportion of λ dimers. This review focuses on the disease-related changes of the structure and level of dimeric FLCs, and raises the questions regarding their formation, function, and role in the pathogenesis and diagnosis of human diseases.

## INTRODUCTION

Human immunoglobulin (Ig) molecules consist of two identical heavy chains and two identical light chains. The latter exist as two isotypes, kappa (κ) or lambda (λ), and they are covalently linked to heavy chains. Production of heavy and light chains, as well as incorporation of light chains into the Ig molecule, occurs in B cells. Normally, light chains are produced in excess over heavy chains. In a healthy state, the majority of light chains in serum is bound to heavy chains, and the serum level of the secreted unbound light chains, i.e., so-called free light chains (FLCs), is low. FLCs can also be detected in urine, cerebrospinal fluid (CSF), and synovial fluid, where the physiological levels of FLCs are also low. Under pathological conditions, however, production of FLCs might be abnormally high[1]. In monoclonal gammopathies, such as multiple myeloma (MM), primary systemic amyloidosis (AL amyloidosis), and nonamyloid light chain deposition disease (NALCDD), the levels of monoclonal FLCs are significantly increased. Also, synthesis of FLCs might be markedly enhanced in certain inflammatory or autoimmune diseases, e.g., multiple sclerosis (MS), systemic lupus erythematosus, AIDS, and Sjogren's disease.

FLC molecules are usually monomers and dimers, but higher polymeric forms might also exist[2,3,4,5]. Under pathological conditions, changes might occur not only in the level, but also in the structure, of monomeric and dimeric FLCs. In AL amyloidosis, structural changes include somatic mutations and post-translational modifications, leading to alterations in the conformation and thermodynamic stability of FLC monomers and dimers[6,7,8,9]. Also, marked increase in the proportion of dimeric and multimeric FLC forms was demonstrated in several diseases, including MM[5,10], AL amyloidosis[11], as well as MS[12]. However, the significance and the role of increased FLC polymerization are still poorly understood. This review focuses on the disease-related changes of the structure and level of dimeric FLCs, and raises the questions regarding their formation, function, and role in pathogenesis and diagnosis of human diseases.

## FORMATION OF FLC DIMERS

Dimeric FLCs are composed of two identical monomeric FLCs (either κ or λ) that bind to each other by covalent or noncovalent links. In the noncovalent dimerization of FLCs, the intermolecular electrostatic, hydrophobic, and hydrogen bonds may play a role. Covalent binding of FLC monomers forming dimers involves an interchain disulfide bond between C-terminal FLC cysteines via oxidation of their thiol (SH) groups acting as electron donors. Lambda □FLCs have a stronger tendency to dimerization (and oligomerization) compared to κ FLCs[3,4].

Conventionally, disulfide bonds have been assumed to confer extra rigidity and stability on their resident protein, but over the last 3 decades, the role of protein disulfides was redefined[13]. It has been found that disulfide bonds are not always stabilizing entities; some disulfides contribute little to stabilization, whereas others destabilize their resident protein. It is now believed that formation of disulfide bonds in proteins occurs intracellulary and is catalyzed by a family of oxireductases. Activity of these enzymes depends on redox potential within different compartments of the cell, thus affecting the ability of the cell to perform oxidation (formation of S-S bridge) or reduction of S-S bond to two thiol groups (SH)[14,15]. Under pathological conditions, such as oxidative stress, a shift in redox potential may occur, thus affecting the subtle balance between dithiol and disulfide states. Of note, oxidative stress markers were found to be elevated in monoclonal gammopathies and autoimmune/inflammatory diseases[16,17,18].

The control of disulfide bond formation is also a part of the quality control machinery within the cell. Such a control is of special interest in protein misfolding diseases, for example, in AL amyloidosis where the production of misfolded FLCs typically occurs. It is now well recognized that misfolded proteins are targeted to proteasomes for degradation. When degradation is imperfect, it may lead to accumulation of misfolded proteins and their degradation products. This, in turn, triggers the unfolded/misfolded protein response, where the up-regulation of folding catalysts (including oxireductases) could stabilize the conformation of misfolded proteins by introducing the disulfide bonds[14,15]. It would be intriguing to find out whether such mechanisms regulate formation of FLC dimers in AL where the abnormally increased level of circulating disulfide-bound plasma FLC species was demonstrated[11].

The noncovalent dimerization of κ and λ FLCs is governed by the amino acid residues present in the framework region of the light chain variable domain. The residues responsible for dimer formation are conserved; tyrosine 36, glutamine 38, and tyrosine 87 take part in interdomain hydrogen bond formation, whereas leucine 46 and phenylalanine 98 form hydrophobic contacts[19]. However, residues from the complementarity determining regions (CDRs) also can affect the exact geometry of the light chain dimer formed[19,20,21]. In fact, dimerization constants ($K_D$) (calculated for 17 Bence Jones proteins) varied significantly, showing difference in $K_D$ of >1000 fold among the κ1 chains[20]. Structural diversity of the variable light chain domain hampers the elucidation of the exact molecular mechanism of FLC dimerization.

Notably, albeit that $K_D$ reflects the degree of noncovalent dimer formation, the conformation of disulfide-bound dimers is affected by the self-affinity indicated by the $K_D$. Generally, λ-type chains (which tend to form covalent dimers) have a higher $K_D$. However, structural reasons for the differences in monomer-monomer interaction in the particular FLC types and subtypes are complex and still not fully understood.

## FUNCTION OF FLC DIMERS IN HUMAN DISEASES

Studies on the biological functions of FLCs demonstrated different biological activities of these proteins, including their capability to modulate the immune system, proteolytic activity[22], complement cascade activation[23], as well as FLC binding to antigens[24,25], chemotactic factors, and opioid peptides[26,27]. Less attention, however, has been given to the analysis of the peculiar functional properties of dimeric FLCs in comparison with their monomeric counterparts. In fact, the structural differences of FLC monomers and dimers imply possible differences in their biological activity. Although the antigen-binding activity of monomeric FLCs was demonstrated in some reports[24,25], it was suggested that due to the structural features of FLC dimers (resembling Fab region of whole Igs[28]), the binding capabilities of dimers may prevail over that of monomers. It was shown that dimeric λ FLCs purified from urine of the MM patient interact with B lymphocytes[29], implying that such interaction might contribute to myeloma-associated immunosuppression. In the other study[30], λ dimers were isolated from serum and urine of a patient with hypocomplementemic membranoproliferative glomerulonephritis; the isolated dimers were found to bind to factor H, the main regulator of alternative pathway of complement. These data suggest that λ FLC dimers might act as pathogenic miniautoantibodies. It was proposed that functional blocking of factor H by an antibody fragment like the λ FLC dimer might initiate the development of a severe form of membranoproliferative glomerulonephritis.

The issues concerning covalent and noncovalent dimerization of FLCs, and the role in pathogenesis of AL amyloidosis, have been discussed in a series of studies[5,7,8,31,32,33,34]. A model for amyloid light chain fibril formation involving dimerization of FLCs was proposed, and the importance of structural alterations of monomer-monomer and dimer-dimer contact regions was highlighted[32]. Analysis of the amyloidogenic light chains demonstrated mutations within the dimer interface; the mutations thought to be responsible for amyloidogenicity[8,9]. The possibility was raised that such mutations may alter dimeric interactions and lead to improper formation of dimers, thus attenuating their thermodynamic stability and promoting amyloidogenicity. These studies call for further analysis of the possible role of dimer formation in AL pathogenesis and would be especially important, as light chain dimer stabilization may be protective against amyloidogenesis.

Of special interest are recent studies showing that FLCs may play a crucial role in the pathogenesis of inflammatory diseases, such as MS[35], rheumatoid arthritis[36], asthma[37,38], rhinitis[39], and inflammatory bowel diseases[40]. It was demonstrated that κ FLCs are capable of sensitizing mast cells, thus leading to mast cell degranulation and *de novo* synthesis and release of inflammatory mediators[41,42,43]. However, it still remains unclear whether FLCs interact with mast cells and, if so, which membrane receptor is involved. A possibility was also raised as to whether dimerization of FLCs is a necessary condition related to cross-linkage of membrane elements[44]. In respect to the latter argumentation, it is worth underscoring the significant elevation of the specific mast cell markers[35,37,45], as well as the increase in the level of FLC dimers in the CSF of MS patients[12], thus indicating a possible link between mast cell activation and enhanced FLC dimerization in MS.

## HIGH LEVEL OF FLC DIMERS IN HUMAN DISEASES

Early studies showed that increased levels of dimeric FLCs might be associated with some of the human diseases. Techniques including double diffusion in agar, immunoelectrophoresis, and gel filtration were applied to study the CSF of patients with subacute sclerosing panencephalitis. It was shown that the abnormal immunoelectrophoretic precipitation line is due to the presence of λ dimers[46]. Further, an abnormally increased λ dimer/monomer ratio was demonstrated in the serum of anephric patients by using gel filtration and radioimmunoassay techniques[3]. Later observations indicated high FLC dimer and multimer levels in AL amyloidosis, MM, and MS.

## Circulating FLC Dimers and Amyloidosis

Over the last 2 decades, much attention has been focused on structural analysis of monoclonal FLCs in AL amyloidosis. Most studies were based on the analysis of excreted urinary FLCs or *in vitro* experiments performed using recombinant wild-type and amyloidogenic light chains. In our study of FLC proteins in plasma of the AL patients[11], we employed Western blotting and mass spectral analysis that added new evidence of the increased level of circulating disulfide-bound FLC species in AL (Fig. 1). The FLC dimerization values in AL amyloidosis were compared with those in two other B-cell proliferative disorders characterized by the presence of monoclonal light chains, i.e., MM and NALCDD. No increase in dimeric FLCs was observed in NALCDD. Our preliminary data showed that the dimer/monomer ratio in MM was patient specific. We assume that the increased disulfide binding of FLC in AL relies on the peculiarities of the FLC structure affected by amyloidogenic mutations and post-translational modifications[6,7,47]. On the other hand, the disease-specific environmental factors within the cell might also affect formation of intermolecular disulfide bonds[14,15].

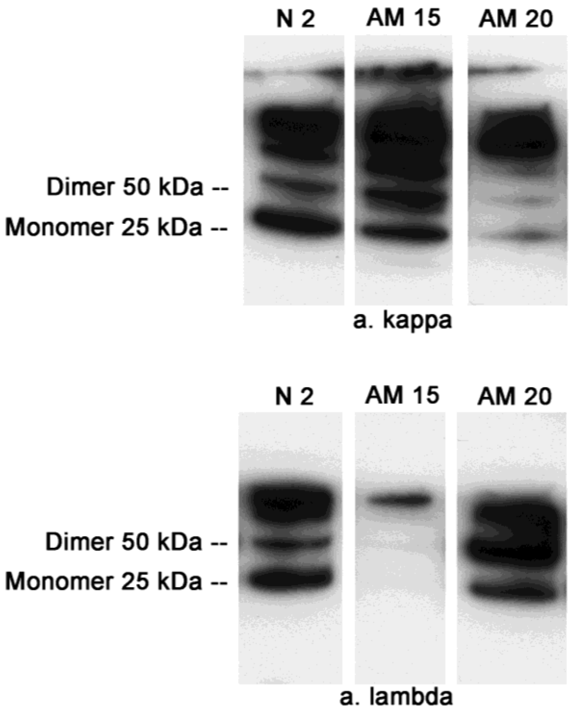

**FIGURE 1.** Western blot analysis of FLCs in the plasma of a healthy person (N2) and patients with AL-κ (AM-15) and AL-λ (AM-20) amyloidoses. Plasma samples were run on 20–25% microgels under nonreducing conditions and immunostained using anti-κ and anti-λ antibodies. Reproduced from Kaplan et al.[11].

The importance of both covalent and noncovalent dimerization of monoclonal FLCs in the formation of fibrillar deposits was highlighted in several reports[7,31,32,33,34]. FLC dimers structurally resemble complete antibodies; therefore, these molecules could bind to extracellular matrix proteins and, as result of these interactions, form amyloid fibrils[48]. This may also explain why λ chains, which are more prone to form dimers than κ chains, are more frequent in AL amyloidosis. In fact, amyloidosis patients with λ light chains outnumber those with κ light chains by a ratio of 3:1. The incidence of λ-type deposits is even higher in renal amyloidosis. The ratio of λ- to κ-type amyloidoses was 4:1 in patients without renal involvement compared to 12:1 in patients with renal amyloidosis[49]. Since the glomerular filtration rate of FLC dimers is lower than that of monomers, the clearance and metabolism of dimeric and multimeric λ dimers is significantly disturbed. Retention of such large multimeric FLC molecules, which are less readily filtered through glomeruli, may lead to their abnormal accumulation and deposition.

Significant progress was made in the understanding of the molecular basis of organ tropism in AL amyloidosis[50]. Renal amyloidosis was commonly associated with the $V_{\lambda 6}$ subtype, while the $V_{\lambda 2}$ and $V_{\lambda 3}$ proteins were typically found in cardiac and soft tissue deposits, respectively. Patients with Vκ clones were more likely to have hepatic involvement[51,52]. Thus, the relationship between VL genes, fibrillogenic light chains, and tissue affinity has important prognostic implications. This also raises a question whether the FLC dimers could act as miniantibodies by recognizing the tissue-specific proteins, and thus contribute to amyloid fibril formation at specific targets[48].

## FLC Dimers and Multiple Myeloma

Early studies demonstrated significantly higher fractions of dimeric FLCs in the serum of MM patients, as compared with that in normal controls[10]. Multimeric FLC complexes were also detected in the serum of patients with MM[5,10,53]. In addition, the tetrameric FLC complexes consisting of two disulfide-bound λ dimers have been described. Another study[5], employing size exclusion chromatography, electrophoresis, analytical ultracentrifugation, and mass spectral analysis, revealed the presence of trimolecular complexes containing λ dimers in the myeloma serum.

MM patients frequently develop renal disease. The major causes of renal failure in MM are cast nephropathy and amyloidosis. Normally, FLCs are filtered across the glomerulus, and completely reabsorbed and metabolized by the proximal tubules. Excessive production of monoclonal FLCs in MM, however, results in the burden of the filtered FLCs exceeding the resorption capacity of the proximal tubules. In cast nephropathy, renal impairment is caused by abnormal accumulation and precipitation of FLCs, which forms casts in the distal tubules, resulting in renal obstruction. Analysis of tissue deposits showed that the eluted monoclonal light chains are often dimers and multimers[54]. As mentioned above, the intrinsic ability of λ FLCs to form dimers and multimers is high compared to that of κ FLCs. This may explain why the MM patients with monoclonal λ-type FLCs may be at higher risk for developing renal dysfunction[52].

## FLC Dimers and Multiple Sclerosis

In our recent study, we applied Western blotting to examine the monomeric and dimeric FLCs in the CSF of MS patients[12]. We found that increased κ monomer and dimer levels, as well as a high κ/λ monomer ratio, were the major FLC parameters typical of MS. In about 40% of MS cases, the elevation of κ FLCs was accompanied by an abnormally high proportion of λ dimers. However, according to our new preliminary data, in some rare MS cases, no significant increase in the κ level was observed, but the λ dimer/monomer ratio was abnormally high (unpublished data) (Fig. 2). No increase in λ dimerization was observed in the sera of MS patients. Further studies are required to understand the significance of these pathological changes occurring during the intrathecal synthesis of light chains and their processing in MS.

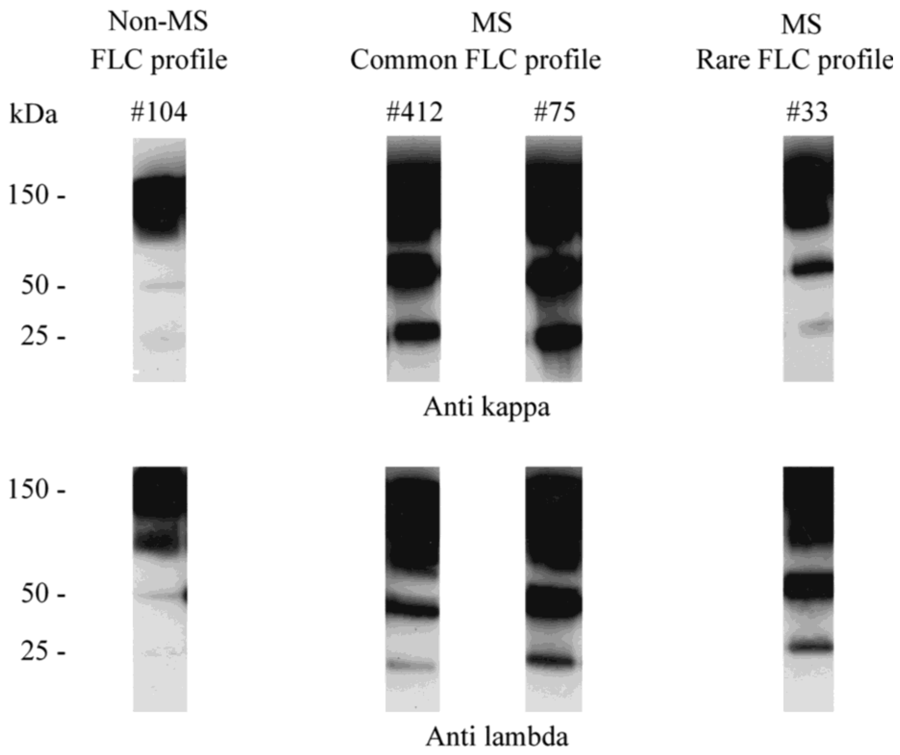

**FIGURE 2.** Western blot analysis of FLCs in the CSF of MS vs. non-MS patients. Samples were run on 10–20% Nu-Sep gels under nonreducing conditions and immunostained using anti-κ and anti-λ antibodies. Typical examples of the common and rare FLC profiles in MS are displayed. The commonly observed FLC profiles (observed in 38 out of 42 MS cases) show the significantly increased levels of κ monomers and dimers, as well as the increase of κ/λ monomer ratios. The level of λ dimers is patient specific. The rare MS profile (observed in four out of 42 MS cases) is characterized by significantly elevated λ dimers, whereas the level of κ and λ monomers was low.

## ABNORMAL DIMERIZATION OF FLCs AND ITS DIAGNOSTIC RELEVANCE

The above-mentioned studies showed that the changes in the structure and level of FLC dimers represent the disease-related pathogenic features that, therefore, may be of diagnostic relevance. In this respect, the development of new methods for detection, characterization, and quantitative evaluation of FLC dimers might be especially helpful. In an attempt to characterize monomeric and dimeric FLC structures, an on-line assay was developed that included immunoaffinity purification with a subsequent electrospray ionization mass spectrometry[34]. The applied method revealed structural heterogeneity of dimeric forms of the amyloidogenic FLCs that were found to be composed of full-length and truncated light chain species. The developed assay may prove helpful to reveal FLC features typical of AL and thus contribute to the diagnosis of this disease. Our study[11] employing the SDS-electrophoresis–based Western blotting technique also showed abnormal heterogeneity of disulfide-bound FLC species in AL. In addition to normally migrating FLC dimers (50 kDa), several aberrantly migrating light chain bands in the 37- to 40-kDa region were observed (Fig. 3). Both the normally and the aberrantly migrating bound FLCs exhibited type-specific immunoreactivity with antibodies to κ (in AL-κ amyloidosis) or to λ light chains (in AL- λ amyloidosis). Fig. 3 demonstrates the prevalence of FLC- λ dimers over FLC-κ dimers in the patients with AL-λ amyloidosis, even in a case where the κ/λ ratio determined by the commonly used nephelometric

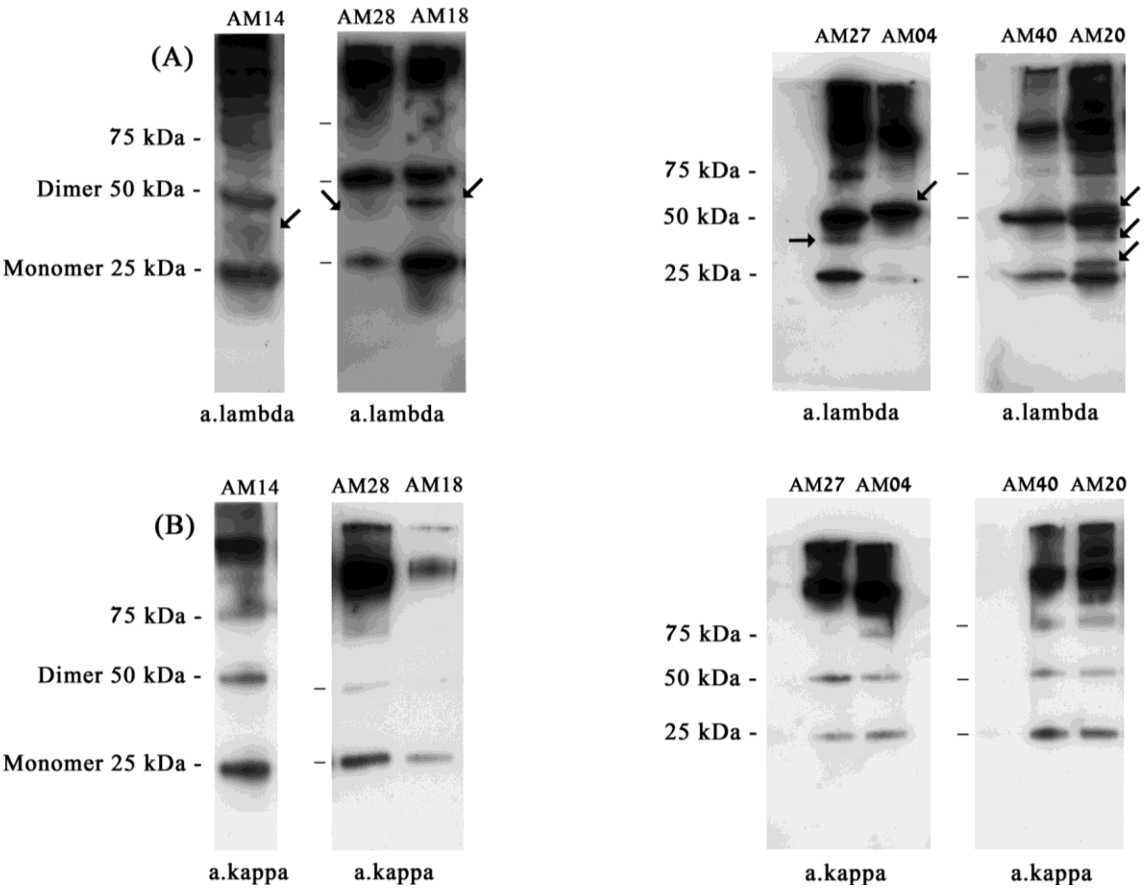

**FIGURE 3**. Western blot analysis of FLCs in the plasma of patients with AL-λ amyloidosis. Plasma samples from patients AM20, AM14, AM28, and AM18 were run on 10–20% Nu-Sep gels under nonreducing conditions and immunostained using anti-κ (A) and anti-λ (B) antibodies. The abnormally migrating monoclonal FLC species are indicated by arrows. Reproduced from Kaplan et al.[11].

FLC assay was normal[14]. The methodological approach applied in this study may be useful for the precise diagnosis of AL amyloidosis by supplementing and confirming the results of the nephelometric FLC assay and the data of the histopathological examination. In this regard, it is worth mentioning that the presence of FLC dimers or oligomers can confound the interpretation of the nephelometric serum FLC assays, which are increasingly used in clinical practice[55]. The nephelometric FLC assay allows an estimation of the total amount of all molecular forms of FLCs (monomers, dimers, and multimers), but the antibodies used in this technique react better with polymeric, rather than monomeric, FLCs. This may result in overestimation of FLC values in samples containing high amounts of dimeric and multimeric forms of FLCs[56,57]. Furthermore, the anti-κ and anti-λ antibodies used in the nephelometric FLC assay were developed against a hidden surface within the intact Ig molecule. Therefore, some epitopes of the hidden region may be unavailable for the antibody due to folding abnormalities of FLCs occurring in the conformational diseases (such as amyloidosis), and thus lead to underestimation of FLC values. For the same reason, these antibodies may not recognize FLC species truncated at the C-termini, whereas the AL serum was shown to contain disulfide-bound monoclonal FLC fragments[11]. These limitations of the nephelometric FLC assay should be taken into consideration, and application of the alternative laboratory techniques, such as immunofixation electrophoresis, might be needed to confirm the obtained data.

Western blot analysis was also used by us to study the FLC monomer-dimer profiles in the CSF and serum of MS patients and those with other neurological diseases[12]. As described above, significant pathological changes in FLC profiles, including high λ dimerization, were observed in the CSF of patients with MS. Analysis of these FLC profiles made possible the selection of most informative FLC parameters to differentiate MS from other neurological diseases. The developed method showed high specificity compared with the conventionally used laboratory method, the oligoclonality test for IgG, and, therefore, may prove to be of promising diagnostic importance.

## CONCLUDING REMARKS

Structural changes in FLC monomers and dimers, as well as increased FLC dimerization, were observed in a number of human diseases related to impaired synthesis of immunoglobulins. The pathophysiological role of these changes remains obscure. However, analysis of monomeric and dimeric forms of FLCs is of diagnostic importance as it might supplement the clinical and laboratory findings and thus contribute to precise diagnosis. Further development and application of new technologies in FLC research may have significant impact in the understanding of pathogenesis, as well in the diagnosis of monoclonal gammopathies and autoimmune/inflammatory diseases.

## References

1. Katzmann, J.A., Clark, R.J., Abraham, R.S., Bryant, S., Lymp, J.F., Bradwell, A.R., and Kyle, R.A. (2002) Serum reference intervals and diagnostic ranges for free kappa and free lambda immunoglobulin light chains: relative sensitivity for detection of monoclonal light chains. *Clin. Chem*. **48**, 1437–1444.
2. Berggard, I. and Peterson, P.P. (1969) Polymeric forms of free normal κ and λ chains of human immunoglobulins. *J. Biol. Chem.* **244**, 4299–4307.
3. Solling, K. (1976) Polymeric forms of free light chains in serum from normal individuals and from patients with renal diseases. *Scand. J. Clin. Lab. Invest*. **36**, 447–452.
4. Solling, K. (1980) Light chain polymerism in normal individuals, in patients with severe proteinuria and in normals with inhibited tubular protein reabsorpsion by lysine. *Scand. J. Clin. Lab. Invest*. **40,** 129–134.
5. Abraham, R.S., Charlesworth, M.C., Owen, B.A.L., Benson, L.M., Katzmann, J.A., Reeder C.B., and Kyle, R.A. (2002) Trimolecular complexes of light chain dimers in serum of a patient with multiple myeloma. *Clin. Chem*. **48**, 1805–1811.
6. Hurle, M.R., Helms, L.R., Li, L., Chan, W., and Wetzel, R. (1994) A role of destabilizing amino acid replacements in light chain amyloidosis. *Proc. Natl. Acad. Sci. U. S. A*. **91**, 5446–5450.
7. Connors, L.H., Jiang, Y., Budnik, M., Theberge, R., Prokaeva, T., Bodi, K.L., Seldin, D.C., Costello, C.E., and Skinner, M. (2007) Heterogeneity in primary structure, post-translational modifications, and germline gene usage of nine full-length amyloidogenic kappa1 immunoglobulin light chains. *Biochemistry* **46**, 14259–14271.
8. Baden, E.M., Owen, B.A.L., Peterson, F.C., Volkman, B.F., Ramirez-Alvarado, M., and Thompson, J.R. (2008) Altered dimer interface decreases stability in an amyloidogenic protein. *J. Biol. Chem.* **283**, 15853–15860.
9. Peterson, F.C., Baden, E.M., Owen, B.A.L., Volkman, B.F., and Ramirez-Alvarado, M. (2010) A simple mutation promotes amyloidogenicity through a highly promiscuous dimer interface. *Structure* **18**, 563–570.
10. Solling, K., Solling, J., and Lanng Nielsen, J. (1984) Polymeric Bence Jones proteins in serum of myeloma patients with renal insufficiency. *Acta Med. Scand*. **216**, 495–502.
11. Kaplan, B., Ramirez-Alvarado, M., Sikkink, L., Golderman, S., Dispenzieri, A., Livneh, A., and Gallo, G. (2009) Free light chains in plasma of patients with light chain amyloidosis and non-amyloid light chain deposition disease. High proportion and heterogeneity of disulfide-linked monoclonal free light chains as pathogenic features of amyloid disease. *Br. J. Haematol*. **144**, 705–715**.**
12. Kaplan, B., Aizenbud, B.M., Golderman, S., Yaskariev, R., and Sela, B. (2010) Free light chain monomers in the diagnosis of multiple sclerosis. *J. Neuroimmunol*. **229**, 263–271.
13. Wouters, M.A., Fan, S.W., and Haworth, N.L. (2010) Disulfides as redox switches: from molecular mechanisms to functional significance. *Antioxid. Redox Signal.* **12**, 53–91.
14. Wilkinson, B. and Gilbert, H. (2004) Protein disulfide isomerase. *Biochem. Biophys. Acta* **1699**, 35–44.
15. Xiao, R., Lundstrom-Ljung, J., Holmgren, A., and Gilbert, H.F. (2005) Catalysis of thio/disulfide exchange *J. Biol. Chem.* **280**, 21099–21106.
16. Migrino, R.Q., Hari, P., Gutterman, D.D., Bright, M., Truran, S., Schlundt, P., and Philips, S.A. (2009) Systemic and microvascular oxidative stress induced by light chain amyloidosis. *Int. J. Cardiol.* **145**, 67–68.
17. Sharma, A., Tripathi, M., Satyam, A., and Kumar, L. (2009) Study on antioxidant level in patients with multiple myeloma. *Leuk. Lymphoma* **50**, 809–815.

18. Ferretti, G., Bacchetti, T., DiLudovico, F., Viti, B., Angeleri, V.A., Danni, M., and Provinciali, L. (2006) Intracellular oxidative activity and respiratory burst of leucocytes isolated from multiple sclerosis patients. *Neurochem. Int*. **48**, 87–92.
19. Schiffer, M. (1996) Molecular anatomy and the pathological expression of antibody light chains. *Am. J. Pathol.* **148**, 1339–1344.
20. Stevens, F.J., Westholm, F.A., Solomon, A., and Schiffer, M. (1980) Self-association of human immunoglobulin kI light chains: role of the third hypervariable region. *Proc. Natl. Acad. Sci. U. S. A.* **77**, 1144–1148.
21. Pokkuluri, P.R., Cai, X., Johnson, G., Stevens, F.J., and Schiffer, M. (2000) Change in dimerization mode by removal of a single unsatisfied polar residue located at the interface. *Protein Sci.* **9**, 1852–1855.
22. Sun, M., Gao, Q.S., Li, L., and Paul, S. (1994) Proteolytic activity of an antibody light chain. *J. Immunol.* **151**, 5121–5126.
23. Meri, S., Koistinen, V., Miettinen, A., Tornroth, T, and Seppala, J.T. (1992) Activation of the alternative pathway of complement by monoclona λ chains in membranoproliferative diseases. *J. Exp. Med*. **175**, 939–950.
24. Mei, S., Lan, L., Qing Sheng, G., and Sudhir, P. (1994) Antigen recognition by an antibody light chain. *J. Biol. Chem*. **269**, 734–738.
25. Masat, L., Wabl, M., and Johnson, J.P. (1994) A simpler sort of antibody. *Proc. Natl. Acad. Sci. U. S. A.* **91**, 893–896.
26. Edmundson, A.B. and Ely, K.R. (1985) Binding of N-formylated chemotactic peptides in crystals of the Mcg light chain dimer: similarities with neutrophil receptors. *Mol. Immunol*. **22**, 463–475.
27. Edmundson, A.B., Ely, K.R., Herron, J.M., and Cheson, B.D. (1985) The binding of opioid peptides to the Mcg light chain dimer: flexible keys and adjustable locks. *Mol. Immunol* **24**, 915–935.
28. Schiffer, G., Girling, R.L., Ely, K.R., and Edmundson, A.B. (1973) Structure of λ-type Bence-Jones protein at 3.5 A resolution. *Biochemistry* **12**, 4620–4631.
29. Wall, J.S., Ayoub, F.M., and O'Shea, P.S. (1996) A study of the interactions of an immunoglobulin λ light chain with artificial and B-lymphocyte membranes. *Front. Biosci.* **1**, a46–58.
30. Jokiranta, T.S., Solomon, A., Pangburn, M.K., Zipfel, P.F., and Meri, S. (1999) Nephritogenic {lambda}light chain dimer: a unique human miniautoantibody against complement factor H. *J. Immunol*. **163**, 4590–4596.
31. Bliznukov, O.P., Kozmin, L.D., Vysotskaya, L.L., Golenkov, A.K., Tishchenko, V.M., Samoylovich, M.P, and Klimovich, V.B. (2005) Human immunoglobulin light chains λ form amyloid fibrils and granular aggregates in solution. *Biochemistry (Moscow)* **70**, 458–456.
32. Shormann, N., Murrel, J.R., Liepnieks, J.J., and Benson, M.D. (1995) Tertiary structure of an amyloid immunoglobulin light chain protein: a proposed model for amyloid fibril formation. *Proc. Natl. Acad. Sci. U. S. A.* **92**, 9490–9494.
33. Stevens, F.J., Myatt, E.A., Chang, C.H., Westholm, F.A., Eulitz, M., Weiss, D.T., Murphy, C., Solomon, A., and Schiffer, M. (1995) A molecular model for self-assembly of amyloid fibrils: immunoglobulin light chains. *Biochemistry* **34**, 10697–10702.
34. Bergen, H.R., III, Abraham, R.S., Johnson, K.L., Bradwell, A.R., and Naylor, S. (2004) Characterization of amyloidogenic immunoglobulin light chains directly from serum by on-line immunoaffinity isolation. *Biomed. Chromatogr*. **18,** 191–201.
35. Zappulla, J.P., Arock, M., Mars, L.T., and Liblau, R.S. (2002) Mast cells: new target for multiple sclerosis therapy? *J. Neuroimmunol.* **131**, 5–20.
36. Lee., D.M., Friend, D.S., Guris, M.F., Benoist, C., Mathis, D., and Brenner M.B. (2002) Mast cells: a cellular link between autoantibodies and inflammatory arthritis. *Science* **296**, 1689–1692.
37. Kraneveld, A.D., van der Kleij, H.P.M., Kool, M., van Houwelingen, A.H., Weitenberg, A.C.D., Redegeld, F.A.M., and Nijkamp, F.P. (2002) Key role for mast cells in nonatopic asthma. *J. Immunol.* **169**, 2044–2053.
38. Kraneveld, A.D., Kool, M., van Houwelingen, A.H., Roholl, P., Solomon, A., Postma, D.S., Nijkamp, F.P., and Redegeld, F.A.M. (2005) Elicitation of allergic asthma by immunoglobulin free light chains. *Proc. Natl. Acad. Sci. U. S. A.* **102**, 1578–1583.
39. Powe, D.G., Kormelink, T.G., Sisson, M., Blokhuis, B.J., Kramer, M.F., Jones, N.S., and Redelgeld, F.A. (2010) Evidence for the involvement of free light chain immunoglobulins in allergic and nonallergic rhinitis. *J. Allergy Clin. Immunol*. **125**, 139–145.
40. Rijnierse, A., Redegeld, F.A., Blokhuis, B.R., Van der Heijden, M.W., Te Velde, A.A., Pronk, I., Hommes, D.W., Nijkamp, F.P., Koster, A.S., and Kraneveld, A.D. (2010) Ig-free light chains play a crucial role in murine mast cell-dependent colitis and are associated with human inflammatory bowel diseases. *J. Immunol*. **185**, 653–659.
41. Rocken, M. and Hultner, L. (2002) Heavy functions for light chains. *Nat. Med*. **8**, 668–670.
42. Redegeld, F.A. and Nijkamp, F.P. (2003) Immunoglobulin free light chains and mast cells: pivotal role in T-cell mediated reactions? *Trends Immunol*. **24**, 181–185.
43. Thio, M., Blokhuis, B.R., Nijkamp F.P., and Redegeld, F.A. (2008) Free immunoglobulin light chains: a novel target in the therapy of inflammatory diseases. *Trends Pharmacol. Sci*. **29**, 170–174.
44. Kaplan, A.P. (2005) A new mechanism for immunologic initiation of asthma. *Proc. Natl. Acad. Sci. U. S. A.* **102,** 1267–1268.
45. Rozniecki, J.J., Hauser, S.L., Stein, M., Lincoln, R., and Theoharides, T.C. (1995) Elevated mast cell tryptase in cerebrospinal fluid of multiple sclerosis patients. *Ann. Neurol*. **37**, 63–66.

46. Riberi, M., Bernard, D., and Depieds, R. (1975) Evidence for the presence of λ chain dimers in cerebrospinal fluid of patients suffering from subacute sclerosing panencephalitis. *Clin. Exp. Immunol.* **19**, 45–53.
47. Buxbaum, J. (1992) Mechanisms of disease: monoclonal immunoglobulin deposition. Amyloidosis, light chain deposition disease, and light and heavy chain deposition disease. *Hematol. Oncol. Clin. North Am.* **6**, 323–346.
48. Solomon, A. (2003) Do $V_L$ genes have prognostic relevance? *Blood* **101**, 3759.
49. Gertz, M.A., Leung, N., Lacy, M.Q., Dispenzieri, A., Zeldenrust, S.R., Hayman, S.R., Buadi, F.K., Dingli, D., Greipp, P.R., Kumar, S.K., Lust, J.A., Rajkumar, S.V., Russell, S.J., and Witzig, T.E. (2009) Clinical outcome of immunoglobulin light chain amyloidosis affecting kidney. *Nephrol. Dial. Transplant.* **24**, 3132–3137.
50. Ronco P. and Aucouturier P. (2009) Renal involvement in AL amyloidosis: the facts, the promise and the hope. *Nephrol. Dial. Transplant.* **24**, 2967–2969.
51. Comenco, R.L., Zhang, Y., Martinez, C., Osman, K., and Herrera, G.A. (2001) The tropism of organ involvement in primary systemic amyloidosis: contributions of Ig VL germ line use and clonal plasma cell burden. *Blood* **98**, 714–720.
52. Abraham, R.S., Geyer, S.M., Price-Troska, T.L., Allmer, C., Kyle, R.A., Gertz, M., and Fonseca, R. (2003) Immunoglobulin light chain variable (V) region genes influence clinical presentation and outcome in light chain-associated amyloidosis (AL). *Blood* **101**, 3801–3808.
53. Grey, H.M. and Kohler, P.F. (1968) A case of tetramer Bence Jones proteinaemia. *Clin. Exp. Immunol.* **3**, 277–285.
54. Santostefano, M., Zanchelli, F., Zaccaria, A., Poletti, G., and Fusaroli, M. (2005) The ultrastructural basis of renal pathology in monoclonal gammopathies. *J. Nephrol.* **18**, 659–675.
55. Bradwell, A.R. (2008) *Serum Free Light Chain Assays*. 5th ed. The Binding Site, Birmingham, U.K.
56. Tate, J., Bazeley, S., Sykes, S., and Mollee, P. (2009) Quantitative serum free light chain assay - analytical issues. *Clin. Biochem. Rev.* **30**, 131–140.
57. Briand, P.Y., Decaux, O., Caillon, H., Grosbois, B., Le Treut, A., and Guenet, L. (2010) Analytical performance of the serum free light chain assay. *Clin. Chim. Lab. Med.*, 73–79.
